# Integrative bioacoustics discrimination of eight delphinid species in the western South Atlantic Ocean

**DOI:** 10.1371/journal.pone.0217977

**Published:** 2019-06-06

**Authors:** Thiago Orion Simões Amorim, Franciele Rezende de Castro, Juliana Rodrigues Moron, Bruna Ribeiro Duque, Juliana Couto Di Tullio, Eduardo Resende Secchi, Artur Andriolo

**Affiliations:** 1 Laboratório de Ecologia Comportamental e Bioacústica - LABEC, Instituto de Ciências Biológicas, Universidade Federal de Juiz de Fora, Juiz de Fora, Minas Gerais, Brazil; 2 Instituto Aqualie, Juiz de Fora, Minas Gerais, Brazil; 3 Laboratório de Ecologia e Conservação da Megafauna Marinha – EcoMega, Instituto de Oceanografia, Universidade Federal do Rio Grande/FURG, Rio Grande, Rio Grande do Sul, Brazil; Sanya Institute of Deep-sea Science and Engineering Chinese Academy of Sciences, CHINA

## Abstract

This study presents an integrative bioacoustics approach to discriminate eight species of odontocetes found on the outer continental shelf and slope of the western South Atlantic Ocean. Spinner, Atlantic spotted, rough-toothed, Risso’s, bottlenose, short-beaked common dolphins, killer and long-finned pilot whales were visually confirmed during recordings with a 3-element omnidirectional hydrophone array. Spectral and time parameters of whistles and echolocation clicks were used in a discriminant function analysis and a classification tree model. As a first step, whistles and clicks were analysed separately; a further analysis consisted of both vocalisations jointly classified. All species showed species-specific properties in their vocalisations. Whistles had greater misclassification rates when compared to clicks. The correct classification was enhanced by the joint step, given the 5.8% error in the discriminant function analysis and a misclassification rate of 18.8% in the tree model. In addition, Receiver Operating Characteristic curves resulting from the tree algorithm analysis exhibited better model efficiency for all species in the joint classification. These findings on acoustical discrimination of such abundant and cosmopolitan species contribute to delphinid classification systems.

## Introduction

Odontocetes species commonly emit tonal frequency-modulated whistles and broadband pulsed clicks and burst sounds [1], and the production patterns of these acoustic signals vary with geographic location, behavioural state, and geometric spacing of conspecifics [2–4].

Some progress has been made in discriminating delphinid whistles to species [5–8] and also with respect to clicks for porpoises, sperm whales, beaked whales and dolphins [9–13]. Although, delphinid whistle and click classifications have received ample focus [14–20], most whistle-based delphinid classifiers have a high misclassification rate.

The interest in interpreting the dolphin sonar system has focused heavily on understanding clicks produced on-axis [11]. For passive acoustic monitoring of free-range odontocetes, on-axis clicks that are acquired may not accurately represent the full variety of clicks [8,11]. It is known that the angle between the longitudinal axis of the odontocete and the hydrophone has acoustical effects on echolocation clicks [11, 20–22]. Therefore, detailed spectral descriptions of stable features and the observed variability across recorded clicks are of interest for wild dolphins, regardless of orientation [20].

The analysis of cetacean sounds at the species level is an important step in processing long-term passive acoustic recordings made in a marine environment [8]. Most studies analysed acoustical emissions separately, examining only click trains [23–25], or whistle sequences [26], or even subunits derived from whistling [27]. However, Rankin *et al*. [28] proposed a compound acoustic classification method for dolphins, which uses whistles, echolocation clicks and burst pulses.

This study proposes an integrative bioacoustics approach using whistles and clicks to discriminate the following eight delphinid species: spinner (*Stenella longirostris*), Atlantic spotted (*S*. *frontalis*), rough-toothed (*Steno bredanensis*), Risso’s (*Grampus griseus*), bottlenose (*Tursiops truncatus*), short-beaked common (*Delphinus delphis*) dolphins, killer (*Orcinus orca*) and long-finned pilot (*Globicephala melas*) whales which are found on the Brazilian outer continental shelf and slope of the western South Atlantic Ocean. There is still no data on the acoustic classification of cetaceans in Brazilian waters.

## Materials and methods

### Study area and data collection

**Fig 1:**
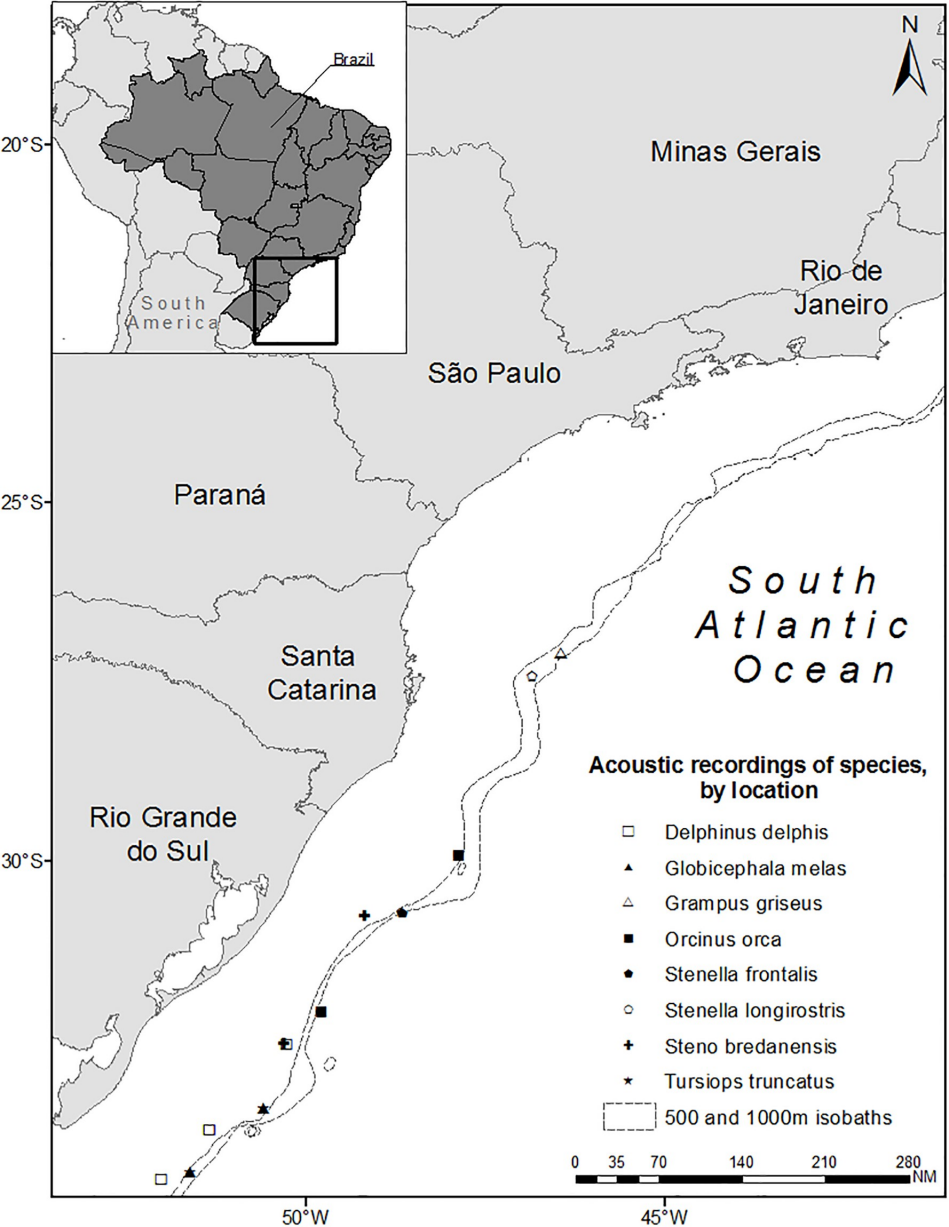
Study area and the acoustic recording locations of each species. Map generated using ArcMap 9.3 (a module of ESRI ArcInfo 9.3, 2006) (http://www.esri.com/).

The present study was conducted opportunistically, during the austral spring and autumn from 2013 to 2015, onboard the R/V Atlântico Sul along the southern and southeastern Brazilian outer continental shelf and slope from Chuí (Rio Grande do Sul State, 33.7° S) to Rio de Janeiro (Rio de Janeiro State, 22.9° S) (Fig 1). Pre-planned zig-zag transect lines, from approximately the 150- to the 1500-metre isobaths, were followed with the vessel’s steering speed varying between 8 and 10 knots. When sea state was below 6 on the Beaufort scale, the area was surveyed during the day (6 am to 6 pm) with constant acoustic recordings and visual monitoring by two observers and at least one experienced support observer. At each sighting, effort was interrupted to approach the sighted group for photographic recording, group size estimation, species identification and acoustic recording. In 65 days of acoustic surveys, the total of recording time was approximately 382 hours. Detailed information of the observation effort is described in Di Tullio *et al*. [29].

The recording system consisted of a 3-element omnidirectional hydrophone array (Auset) coupled to a digital recorder (Fostex FR-2 LE). The sampling rate adopted was 96 kHz and 48 kHz/24 bits. For acoustic analysis, only one channel and recordings of groups that were observed to contain a single species were considered. Table 1 summarizes the data used for analysis and equipment information, and Fig 1 shows the recording locations of each species.

**Table 1:** Overview of data used in the analysis, including the date of recordings, number of sightings/groups, time of recordings, and equipment information.

Species	Date	Number of sightings/groups	Group size	Recording time	Recording duration (min)	Recording coordinates		Sample rate (kHz)^a^	High pass filter (Hz)
						Latitude	Longitude		
*Delphinus delphis*	8 May 2014	4	80	2:05pm	83	-33.768	-51.356	48	1592
	13 May 2014		230	5:13pm	88	-32.565	-50.275		
	12 November 2014		110	10:48am	52	-34.458	-52.014	96	499
	14 November 2014		250	10:50am	17	-33.766	-51.356		
*Globicephala melas*	12 May 2014	2	60	08:05am	165	-33.464	-50.585	48	1592
	12 November 2014		45	4:10pm	35	-34.357	-51.62	96	499
*Grampus griseus*	04 June 2013	1	230	07:06am	159	-27.105	-46.44	96	1592
*Orcinus orca*	24 May 2013	2	15	09:10am	185	-29.932	-47.86	96	1592
	21 November 2014		8	05:52am	56	-32.115	-49.78		499
*Stenella frontalis*	22 May 2013	1	^b^	05:39am	37	-30.726	-48.639	96	1592
*Stenella longirostris*	03 June 2013	1	400	08:29am	38	-27.41	-46.834	96	1592
*Steno bredanensis*	25 November 2014	2	20	4:38pm	57	-30.77	-49.172	96	499
	18 May 2015		50	2:46pm	55	-32.546	-50.299		
*Tursiops truncatus*	12 May 2014	2	70	08:05am	165	-33.464	-50.585	48	1592
	14 June 2013		^b^	1:31pm	9	-22.907	-42.025	96	

^a^ Whistle analysis used files at 48 kHz and 96 kHz. Differences in sample rates did not affect the discriminant results, given that the maximum frequency of whistles did not exceed the Nyquist frequency for both cases. Clicks were only analyzed in files with 96 kHz of sampling rate.

^b^ Not possible to estimate the group size due to bad sighting conditions.

The acoustic monitoring was conducted passively; it did not require authorization from ethics committee. The study area of this work is located in the Brazilian Economic Exclusive Zone and, which did not include any protected area. Therefore, no specific permission was required.

### Signal processing

#### Whistle analysis

Recordings of cetaceans that had been visually identified to the species level were analysed using Raven Pro 1.5 (Cornell Laboratory of Ornithology, NY). The spectrograms were aurally inspected, and sequences were selected out of all recordings that showed high signal to noise ratio (at least 10 dB). Only whistles that were clear in overall contour shape and did not overlap with other whistles were randomly chosen for analysis. The following parameters were extracted from the spectrograms (Hamming window of 1024 points of FFT with 60% overlap): maximum frequency, minimum frequency, delta frequency (maximum frequency—minimum frequency), peak frequency, centre frequency, beginning frequency, ending frequency, and duration. The whistles of killer whales and spinner dolphins were previously reported in Andriolo *et al*. [30] and Moron *et al*. [31] respectively.

#### Click analysis

First, the spectrograms were visually inspected (sample rate of 96 kHz, Hamming window of 256 points of FFT with 50% overlap, resolution of 2.67 ms in time and 188 Hz in frequency) and the echolocation trains were visually detected. Second, subsets of data were created with each audio file containing only one click train from an identified species. A high pass filter with a cut-off at 10 kHz was applied to minimize the influence of low frequency noise. All selected echolocation signals, independent of the recorded animal’s angle to the hydrophone array, were included in the analysis.

The subsets of recording files were analysed using a custom routine in MATLAB (Mathworks, Natick, MA). The following acoustic parameters were extracted: inter-click interval (ICI), 3 dB bandwidth, and 10 dB bandwidth. In addition, for ICI measurements, only signals, which could be aurally assigned to one vocalizing animal and which did not show any other clicks belonging to a different train, were considered. This step was important for avoiding overlapped click trains, which underestimate the real ICI value (S1 Fig). For click analysis, the acoustic system was configured to provide an anti-aliasing filter and because of the cut-off limit of the equipment, peak frequency and centre frequency were not measured.

### Statistical analysis

#### Discriminant function analysis

Prior to a discriminant function analysis (DFA), Kruskal-Wallis’ one-way analyses of the variance were applied to whistle and click parameters for species discrimination. A post-hoc Dunn test with Bonferroni correction was run to single out non-significant results among pairs of species.

Then, a DFA was performed with the software JMP 12 (SAS Institute Inc., Cary, NC) given that the following assumptions were met: (1) Lack of incongruent observation or outliers: outliers were removed since they can affect normality and homogeneity of variance; (2) Normality: a Shapiro-Wilk test was used to evaluate normality and data were log transformed if necessary; (3) Multicollinearity: a correlation matrix was used to verify the multicollinearity among the variables. A Spearman correlation was then applied to check the association among the acoustic parameters. Highly correlated acoustic parameters were not considered in the DFA, since the predictive power can decrease with an increased correlation between predictor variables; (4) Homoscedasticity: a Bartlett test for the equality of variances/covariance was applied. Quadratic discriminant analysis was used when the covariances were not equal; (5) Independence: vocalizations were randomly sampled. To ensure the independence of data and to avoid oversampling groups or individuals, a maximum of 100 randomly selected whistles were analysed from each recording session. For clicks, the data were reduced to avoid over representation of the same individual signal and to maintain independence of the clicks analysed. At least 20 click trains for each species were randomly selected from the beginning to the end of a recording (for the total number of clicks see Table 2). Then, from each sampled train, a limited number of clicks were selected at random. The Entropy RSquare was calculated as a measure of fit [32]. The level of significance adopted for all tests was α = 0.05.

**Table 2:** Spectral and temporal parameter of whistles and clicks for all species given as the median with the 10th and 90th percentile in parentheses. MinF: minimum frequency, MaxF: maximum frequency, DeltaF: delta frequency, PeakF: peak frequency, CentreF: centre frequency, BeginF: beginning frequency, EndF: ending frequency, Duration, ICI: inter-click interval, 3 dB bw: 3 dB bandwidth, 10 dB bw: 10 dB bandwidth. Number of analyzed vocalizations is given as N: number of whistles/number of clicks.

Species	Whistles								Clicks		
	MinF	MaxF	DeltaF	PeakF	CentreF	BeginF	EndF	Duration	ICI	3 dB bw	10 dB bw
	(kHz)	(kHz)	(kHz)	(kHz)	(kHz)	(kHz)	(kHz)	(ms)	(ms)	(kHz)	(kHz)
*Delphinus delphis*	8.6	15.3	5.5	11.6	12.4	11.7	13	735	51.7	6.3	24.2
(N: 202/397)	(5.6–12.9)	(10.7–20.2)	(1.8–10.3)	(8.3–17.4)	(8.4–17.9)	(7.5–17.1)	(6.8–17.0)	(303.0–1270.0)	(32.7–78.0)	(6.1–6.5)	(11.2–35.5)
*Globicephala melas*	8.8	14	4.1	10.9	11.1	11	12.3	460	294.1	12.3	28.1
(N: 117/101)	(2.8–13.3)	(4.1–19.2)	(1.02–9.6)	(3.5–17.8)	(3.6–17.6)	(3.5–17.6)	(4.0–17.4)	(162.0–978.0)	(30.5–392.4)	(8.3–33.4)	(13.5–37.7)
*Grampus griseus*	7.1	15.4	7.7	11.1	11	10.7	9.7	690	131.3	9.6	25.7
(N: 247/340)	(1.8 1–11.2)	(3.1–18.7)	(1.0–11.7)	(2.6–15.5)	(2.2–14.5)	(2.6–14.8)	(2.1–15.9)	(220.0–1390.0)	(43.7–442.4)	(7.2–15.3)	(15.8–38.7)
*Orcinus orca*	3.9	8.2	4.1	5.6	7.1	4.3	8	295	28.2	16	14.8
(N: 70/187)	(1.9–15.1)	(3.8–24.6)	(1.6–9.3)	(2.7–17.8)	(3.3–20.3)	(1.9–24.5)	(3.4–16.9)	(110.0–838.0)	(21.9–49.3)	(8.2–31.6)	(11.5–32.3)
*Stenella frontalis*	9.6	16.9	7.7	15.4	14.5	9.9	16.8	320	52.2	12.9	31.1
(N: 98/892)	(5.4–11.9)	(15.8–19.6)	(4.5–12.9)	(12.2–17.1)	(13.3–15.5)	(5.7–12.7)	(15.4–19.4)	(212.8–499.5)	(26.7–105.7)	(9.4–30.0)	(18.7–38.6)
*Stenella longirostris*	9.9	15.9	4.6	13.8	13.5	11.8	13.7	690	100.7	19.4	26.2
(N: 147/768)	(6.5–15.1)	(10.5–20.9)	(1.3–9.4)	(9.1–16.7)	(9.4–16.3)	(6.8–17.3)	(8.6–18.7)	(200.0–1416.0)	(51.9–232.8)	(10.9 37.5)	(20.4–34.2)
*Steno bredanensis*	5.8	8.1	2.2	7.4	6.9	6.3	7.6	409	55.2	19	32
(N: 113/470)	(4.0–8.3)	(6.7–10.1)	(0.5–4.9)	(5.6–8.8)	(5.5–8.9)	(4.1–8.8)	(5.8–9.9)	(126.6–974.4)	(21.9–167.2)	(9.3–35.7)	(20.8–39.1)
*Tursiops truncatus*	9.7	16.2	5.6	12.7	12	12.6	13.8	370	37.1	30.5	24.5
(N: 301/1005)	(7.2–14.6)	(12.5–19.7)	(1.9–9.8)	(9.1–17.9)	(9.9–17.9)	(8.7–16.4)	(8.3–17.6)	(140.0–966.0)	(23.8–71.7)	(13.4–34.4)	(22.4–29.4)

In order to verify the accuracy of the DFA in classifying the species, whistles and clicks were first tested separately, then in a joint analysis. This step consisted of input the merged vectors of logarithmized parameters of both vocalizations (whistles and clicks) in the models, which analysed them jointly as explanatory variables of each species regardless the type of vocalization these parameters refer to. For each of these steps, the DFA assumptions were re-evaluated.

#### Classification tree algorithm analysis

Classification tree analysis was performed using JMP 12. This method is a non-parametric classification technique, in which a tree is grown by separating data into groups through sequential binary partitioning (splits) of the predictor variables. Unlike DFA, classification trees are not sensitive to outliers and are tolerant of observations with missing values [32].

As was done with the DFA procedure, whistles and clicks were first tested separately, then both were analysed jointly to verify whether the combined vocalisations were more efficient in the discrimination. Finally, Receiver Operating Characteristic (ROC) curves were created in order to verify the classifying efficiency of the model, and the G^2^ (Likelihood-ratio Chi-square) categorical statistics and the number of splits were computed to inspect the contribution of each input parameter to the model. The misclassification rates were calculated using k-fold cross validation (k = 10) and the RSquare values were computed.

## Results

### Differences among acoustic parameters of whistles and clicks of delphinid species

When tested for differences with a Kruskal-Wallis test, all parameters for clicks and whistles showed significant results (p<0.05) for all species (S1 Table). The click parameter inter-click interval (Chi^2^ = 1470.4) and the whistle ending frequency (Chi^2^ = 347.0) showed the highest significance levels.

The overview of descriptive data for each species is presented in Table 2.

Short-beaked common dolphins had the lowest median value for the 3 dB bandwidth and the highest whistle duration. Risso’s dolphins presented the lowest median values for inter-click interval and the highest value for whistle delta frequency. Killer whales had lower values for minimum frequency, peak frequency, beginning frequency, whistle duration and 10 dB bandwidth. Atlantic spotted dolphins had the highest values for maximum frequency, delta frequency, peak frequency, centre frequency and ending frequency, while spinner dolphins had the highest median values for minimum frequency and inter-click interval. Rough-toothed dolphins showed the lowest values for maximum frequency, delta frequency, centre frequency, and ending frequency, and the highest value for 10 dB bandwidth. Finally, bottlenose dolphins presented the highest values for beginning frequency and 3 dB bandwidth.

### Integrative acoustic discrimination among delphinid species

#### Discriminant function analysis

Assuming equal prior probabilities among species, the DFA showed that whistle classification had the highest number of false classifications (40.7%, N = 475, Wilks’ λ = 0.18, p<0.0001, Entropy RSquare = 0.44); these numbers decreased when only clicks were taken into account (25.0%, N = 158, Wilks’ λ = 0.14, p<0.0001, Entropy RSquare = 0.69). The discrimination result improved with the joint analysis of whistles and clicks, given that the misclassification percentage was 5.8 (N = 30, Wilks’ λ = 0.01, p<0.0001, Entropy RSquare = 0.92) (Table 3). Discriminant scores reports of whistles, clicks and joint analysis providing the predicted classification of each observation are presented in S2, S3 and S4 Tables respectively.

**Table 3:** Confusion matrix of discriminant function analysis. Entropy RSquare, Percentage and number of misclassifications are presented.

**Whistles**									
	Dd	Gg	Gm	Oo	Sb	Sf	Sl	Tt	Total
Dd	45.4	16.9	0.0	0.0	3.3	2.2	20.8	11.5	183
Gg	14.9	50.6	0.0	0.0	0.0	2.9	9.2	22.4	174
Gm	1.8	0.9	67.9	3.6	25.0	0.0	0.0	0.9	112
Oo	0.0	1.4	2.9	89.9	1.4	0.0	0.0	4.3	69
Sb	0.9	0.0	4.7	4.7	88.8	0.0	0.0	0.9	107
Sf	0.0	1.3	0.0	0.0	0.0	96.3	0.0	2.5	80
Sl	11.5	7.9	0.0	1.4	4.3	1.4	55.4	18.0	139
Tt	15.3	13.0	0.0	2.3	4.3	4.3	16.9	43.9	301
Entropy RSquare = 0.44									
Percent misclassified = 40.7									
Number misclassified = 475									
**Clicks**									
	Dd	Gg	Gm	Oo	Sb	Sf	Sl	Tt	Total
Dd	93.8	6.3	0.0	0.0	0.0	0.0	0.0	0.0	64
Gg	0.0	98.9	0.0	0.0	1.1	0.0	0.0	0.0	89
Gm	0.0	3.0	54.5	12.1	24.2	3.0	3.0	0.0	33
Oo	0.0	0.0	0.0	89.4	3.8	6.7	0.0	0.0	104
Sb	0.0	11.4	2.3	12.5	60.2	6.8	2.3	4.5	88
Sf	0.0	0.0	4.4	3.3	3.3	31.9	12.1	45.1	91
Sl	0.0	0.0	0.0	0.0	1.2	9.9	74.1	14.8	81
Tt	0.0	0.0	0.0	2.4	0.0	6.1	2.4	89.0	82
Entropy RSquare = 0.69									
Percent misclassified = 25.0									
Number misclassified = 158									
**Whistles + clicks**^a^									
	Dd	Gg	Gm	Oo	Sb	Sf	Sl	Tt	Total
Dd	100.0	0.0	0.0	0.0	0.0	0.0	0.0	0.0	55
Gg	0.0	100.0	0.0	0.0	0.0	0.0	0.0	0.0	44
Gm	0.0	0.0	83.9	0.0	16.1	0.0	0.0	0.0	31
Oo	0.0	0.0	0.0	100.0	0.0	0.0	0.0	0.0	69
Sb	0.0	0.0	3.6	0.0	95.2	0.0	1.2	0.0	83
Sf	0.0	0.0	0.0	0.0	0.0	95.7	1.4	2.9	70
Sl	0.0	0.0	0.0	0.0	2.5	0.0	86.3	11.3	80
Tt	0.0	0.0	0.0	1.2	1.2	1.2	4.9	91.5	82
Entropy RSquare = 0.92									
Percent misclassified = 5.8									
Number misclassified = 30									

Dd: *Delphinus delphis*, Gm: *Globicephala melas*, Gg: *Grampus griseus*, Oo: *Orcinus orca*, Sf: *Stenella frontalis*, Sl: *Stenella longirostris*, Sb: *Steno bredanensis*, Tt: T*ursiops truncatus*

^a^ Joint analysis

**Fig 2:**
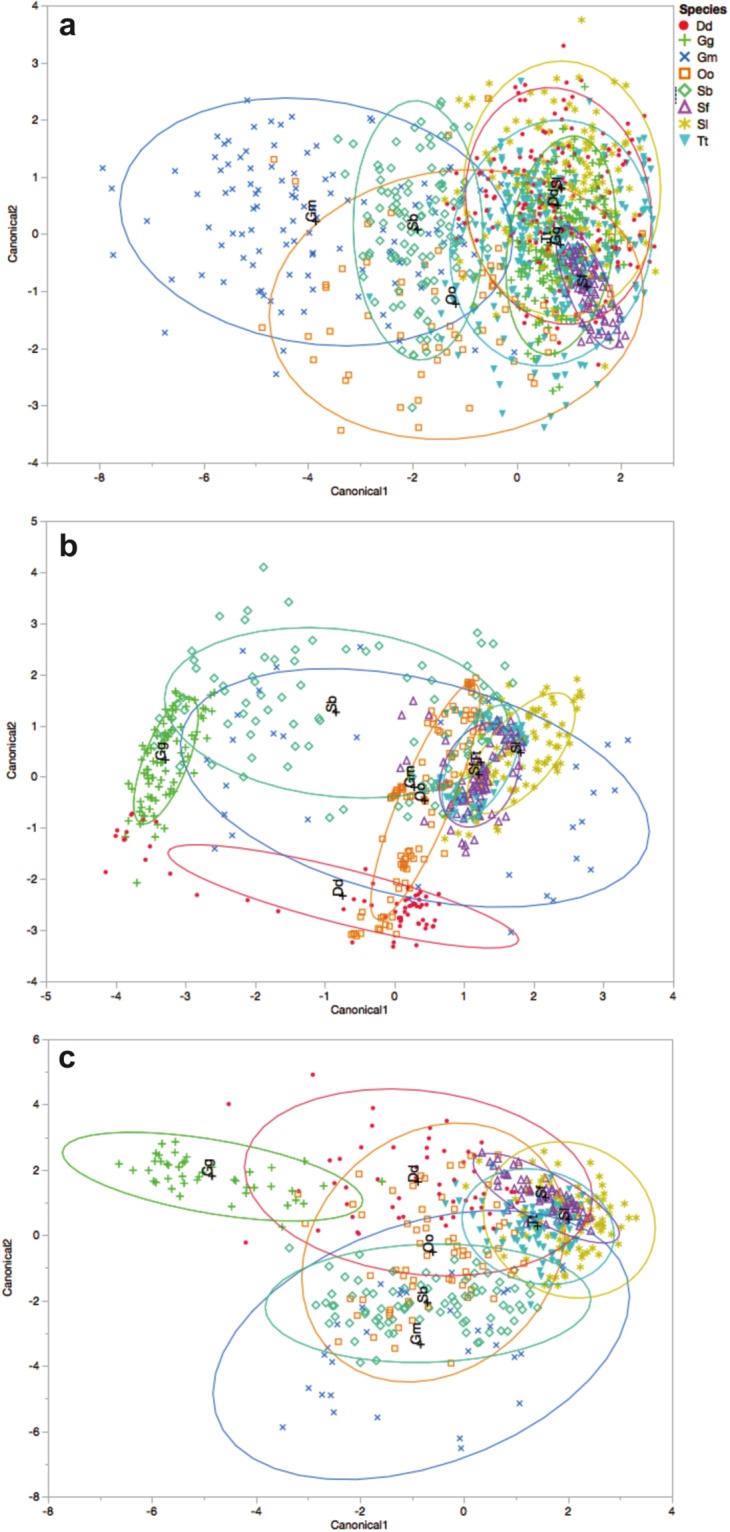
Discriminant function analysis results represented in multivariate space. (A) Whistle classification, PC1 = 81.98% and PC2 = 91.66% (cumulative percentage); (B) click classification, PC1 = 74.03% and PC2 = 99.13% and (C) whistle and click classification, PC1 = 46.17% and PC2 = 74.75%. Ellipses contain approximately 50% of the observations for each species. Dd: *Delphinus delphis*, Gm: *Globicephala melas*, Gg: *Grampus griseus*, Oo: *Orcinus orca*, Sf: *Stenella frontalis*, Sl: *Stenella longirostris*, Sb: *Steno bredanensis*, Tt: *Tursiops truncatus*.

Within species, the DFA showed that whistle classification had the highest percentage of correct classification for killer whales (89.9%), Atlantic spotted dolphins (96.3%) and rough-toothed dolphins (88.8%). For clicks, short-beaked common dolphins (93.8%), Risso’s dolphins (98.9%) and bottlenose dolphins (89.0%) had the highest percentage of correct classification. The discrimination result improved in the joint classification. Common dolphins, Rissos’s dolphins and killer whales were classified with 100% correct discrimination (Table 3). Plots for the first two canonical discriminant functions are shown in Fig 2.

#### Classification tree analysis

Using all acoustic parameters, the optimal classification whistle tree consisted of 28 splits and a misclassification rate of 60.6% (RSquare = 0.23). The optimal click tree consisted of 60 splits and a misclassification rate of 26.0% (RSquare = 0.63). The optimal tree of the joint analysis consisted of 90 splits and a false classification of 18.8% RSquare = 0.71) (Table 4).

**Table 4:** Confusion matrix of tree algorithm analysis. Number of observations and misclassifications (M.R) are presented.

**Whistles**									
	Dd	Gm	Gg	Oo	Sf	Sl	Sb	Tt	Total
Dd	10.9	5.4	8.7	0.0	0.0	14.1	4.3	56.5	92
Gm	0.0	30.4	1.8	1.8	0.0	14.3	5.4	46.4	56
Gg	8.3	5.0	41.7	0.0	0.0	5.0	3.3	36.7	60
Oo	0.0	0.0	3.3	26.7	0.0	0.0	0.0	70.0	30
Sf	0.0	0.0	0.0	0.0	4.6	1.0	0.0	94.4	196
Sl	0.0	1.2	0.0	0.0	0.0	10.2	1.2	87.3	166
Sb	0.0	0.0	1.1	0.0	0.0	1.1	20.5	77.3	88
Tt	0.0	3.3	0.0	0.0	0.0	0.9	0.5	95.3	212
M.R = 60.6%									
**Clicks**									
	Dd	Gm	Gg	Oo	Sf	Sl	Sb	Tt	Total
Dd	89.7	10.0	0.3	0.0	0.0	0.0	0.0	0.0	311
Gm	0.0	88.8	0.5	1.9	0.9	0.5	6.5	0.9	214
Gg	0.0	19.2	65.1	0.0	11.0	4.8	0.0	0.0	292
Oo	1.4	7.6	1.4	48.3	17.2	1.4	7.6	15.2	145
Sf	0.0	0.0	3.8	0.0	61.1	2.4	4.1	28.6	740
Sl	0.0	0.0	2.2	0.3	7.5	78.6	8.1	3.3	630
Sb	0.3	0.0	7.8	2.3	14.3	17.3	42.5	15.8	400
Tt	0.0	0.0	2.6	0.0	4.4	0.8	0.9	91.3	1003
M.R = 26.0%									
**Whistles + clicks**^a^									
	Dd	Gm	Gg	Oo	Sf	Sl	Sb	Tt	Total
Dd	90.5	0.0	9.5	0.0	0.0	0.0	0.0	0.0	316
Gm	0.0	95.7	0.8	1.2	0.0	0.4	2.0	0.0	253
Gg	0.7	0.4	90.2	0.0	4.3	2.2	0.7	1.4	276
Oo	1.1	0.0	6.3	55.2	17.8	5.2	2.3	12.1	174
Sf	0.0	0.0	0.0	0.1	73.7	2.5	4.6	19.0	673
Sl	0.0	0.0	0.2	0.0	4.9	86.0	5.6	3.3	629
Sb	0.3	0.0	0.0	1.7	15.4	11.0	56.8	14.8	345
Tt	0.0	0.0	0.2	0.0	4.1	0.4	0.6	94.8	1012
M.R = 18.8%									

Dd: *Delphinus delphis*, Gm: *Globicephala melas*, Gg: *Grampus griseus*, Oo: *Orcinus orca*, Sf: *Stenella frontalis*, Sl: *Stenella longirostris*, Sb: *Steno bredanensis*, *Tt*: *Tursiops truncatus*

^a^ Joint analysis

Correct classification scores for whistles of individual species ranged from 4.6% (Atlantic spotted dolphin) to 95.3% (bottlenose dolphin). For clicks, the percentage of correct classification was higher, ranging from 42.5% (rough-toothed dolphin) to 91.3% (bottlenose dolphin). Correct classification scores for the whistle and click joint analysis ranged from 55.2% (killer whale) to 95.7% (long-finned pilot whale) (Table 4).

**Fig 3:**
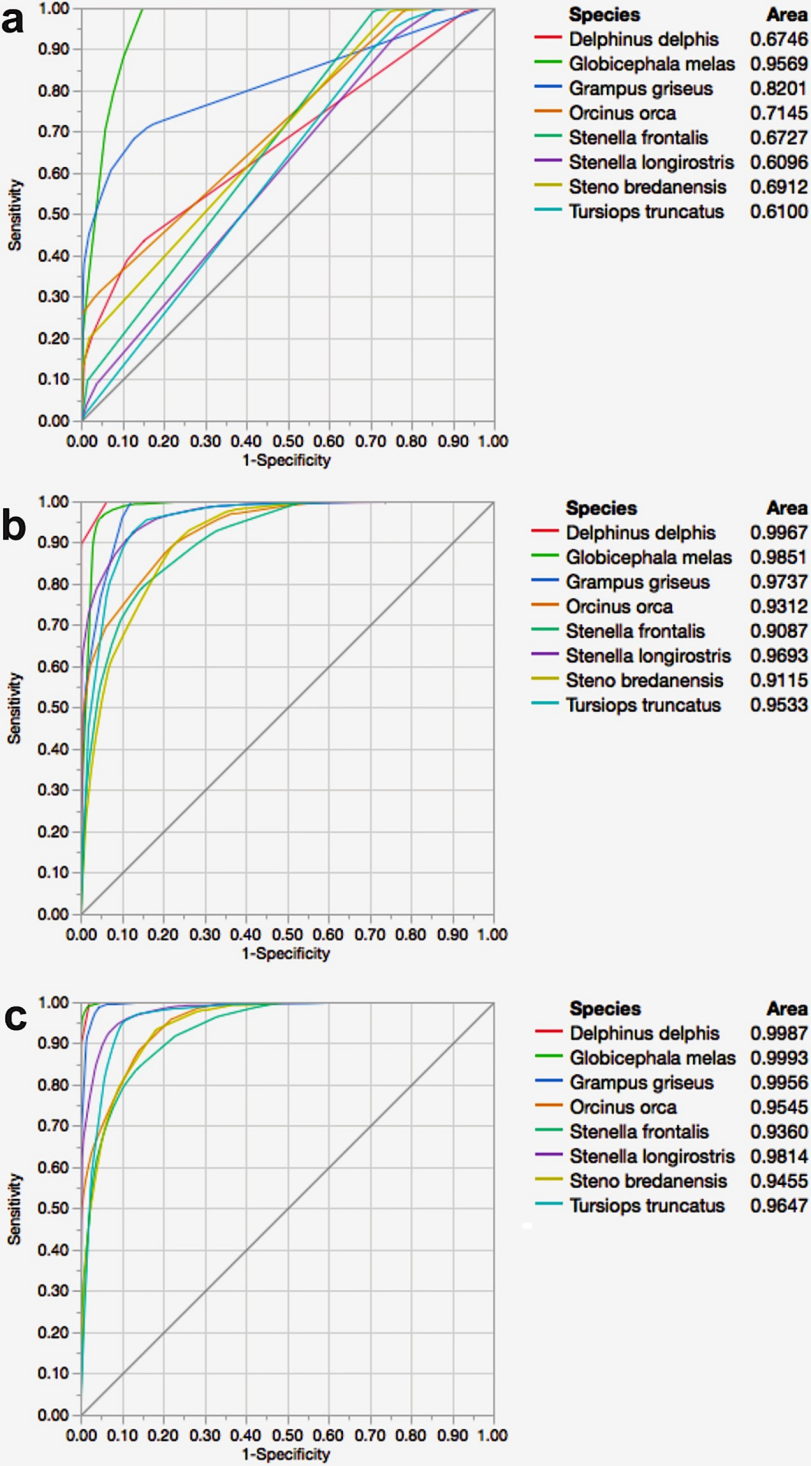
Receiver Operating Characteristic (ROC) curves. Each curve represents the sorting of the efficiency of the model for all species. The area under the curve is the indicator of the goodness of fit; a value of 1 indicates a perfect fit. The classical definition of ROC curves involves the count of True Positives by False Positives as the frequencies across a rank ordering are accumulated. The True Positive Y-axis is labelled “Sensitivity” and the False Positive X-axis is labelled “1-Specificity”. (A) Whistle analysis, (B) click analysis and (C) whistle and click analysis.

The ROC curve of whistles showed that long-finned pilot whales best fitted the model (area = 0.9569) and spinner dolphins had a lower efficiency fit with an area of 0.6096 (Fig 3A). The goodness of fit increased when clicks were analysed. Long-finned pilot whale best fitted the model with an area of 0.9851 and Atlantic spotted dolphin had the lower area of 0.9087 (Fig 3B). The ROC curve of the joint classification showed that long-finned pilot whale best fitted the model with an area of 0.9993 and Atlantic spotted dolphin had the smallest area (0.9360) (Fig 3C).

Whistle contribution parameter analysis showed that the maximum frequency contributed greatly to the model (G^2^ = 1524.89) and the beginning frequency was the least important with a G^2^ value of 95.93. The delta frequency did not contribute to the discrimination of whistles using the tree model. The 3 dB bandwidth was the parameter that most contributed to the click tree (G^2^ = 5065.64), whereas the 10 dB bandwidth was the least important with a G^2^ of 699.95. When whistles and clicks were analysed together, the 3 dB bandwidth contributed the most to discrimination (G^2^ = 4977.24) and duration was the parameter that contributed the least (G^2^ = 25.20) (Table 5).

**Table 5:** Parameter contributions, G2 (Likelihood-ratio Chi-square) categorical statistics and the number of splits in discriminant tree model. MinF: minimum frequency, MaxF: maximum frequency, DeltaF: delta frequency, PeakF: peak frequency, CentreF: centre frequency, BeginF: beginning frequency, EndF: ending frequency, Duration, ICI: inter-click interval, 3 dB bandwidth, 10 dB bandwidth.

Whistles			Clicks			Whistles + clicks^a^		
Parameters	Number of splits	G^2^	Parameters	Number of splits	G^2^	Parameters	Number of splits	G^2^
MaxF	4	1524.89	3 dB bandwidth	28	5065.64	3 dB bandwidth	33	4977.24
PeakF	5	672.97	ICI	20	3530.64	ICI	24	3546.21
Duration	5	271.07	10 dB bandwidth	12	699.95	DeltaF	1	548.34
MinF	4	256.22				10 dB bandwidth	8	528.42
CentreF	5	193.13				PeakF	6	452.75
EndF	3	172.02				MaxF	4	182.57
BeginF	2	95.93				CentreF	5	154.59
DeltaF	0	0.00				EndF	4	99.89
						MinF	3	46.06
						BegginF	1	32.66
						Duration	1	25.20

^a^ Joint analysis

## Discussion

Whistle and click parameters were significantly different among species considering the Kruskal-Wallis test, which was important to determine if such parameters were eligible for the further discriminant analysis (e.g. [8]), then used all parameters. Unlike Kruskal-Wallis test that compare parameter-by-parameter, the discriminant analysis distinguishes the species from the result of an interaction among the acoustical parameters. Therefore, the observed significance for Kruskal-Wallis is not necessarily associated to a clear separation among species, since the discrimination is given by an interaction of parameters and not an individual analysis of each one.

The results of both the DFA and the classification tree analysis suggest that whistles and clicks may be suitable for delphinid species identification. Overall, whistle misclassification was 40.7% in the DFA. Based on the whistle parameter, killer whales, rough-toothed dolphins and Atlantic spotted dolphins have the most distinctive whistles. The correct association of whistles with species may be determined by group composition, distinctive individual vocal characteristics, and relative contribution in social contexts [14,33]. Therefore, it is likely that the degree of differences scored in the whistle vocalisations among the eight species may have a behavioural component, which could have resulted in higher misclassification scores compared to clicks.

Considering clicks, the DFA misclassification score of 25.0% was considerably lower than the whistle score. Animal morphology, particularly anatomical head structure, such as skull morphology and sound producing organs, may have an influence on the spectral and temporal patterns of clicks, and therefore, could be a relevant factor for acoustic discrimination of a species [11,8]. Spectral and temporal parameters of echolocation clicks differ largely with the orientation of the animals’ heads relative to the hydrophone [33,34]. Considering the field methods, it was assumed that most clicks described were recorded off-axis. According to Soldevilla *et al*. [11] and Roch *et al*. [35] the sound reverberation inside the animals’ heads, particularly in off-axis click emissions, can carry species-specific information.

The results of both the DFA and the classification tree analysis suggest that using clicks is a reliable tool for species discrimination. This also relies on the fact that all species grouped together more clearly on the canonical plots (Fig 2A and 2B), resulting in higher correct classification scores. Aside from that, descriptions of acoustical parameters (Table 2), considering all variability, are of interest in studies of free-ranging species in their natural habitats. Thus, not only clicks, but also whistle classification should be able to incorporate variability and still be able to reliably identify to species level.

Overall correct classification rates from the DFA (a parametric statistical method) and the classification tree model (a non-parametric method) supports the use of either technique for species identification. In both analyses, correct classification scores were higher when whistles and clicks were jointly classified (Fig 2C, Tables 3 and 4). The values of entropy RSquare and the areas of ROC curves corroborated that the joint analysis increases the efficiency of the models.

Likelihood-ratio Chi-square (G^2^) of parameter contributions to the model (Table 5) showed that maximum frequency had a greater contribution to whistle analysis, whereas 3 dB bandwidth was the parameter that most contributed to the click tree, suggesting that these two variables may be important for species differentiation. The 3 dB bandwidth was still the parameter with a higher G^2^ when whistles and clicks were joint classified, thus it was the variable that contributed the most in the integrative analysis of the tree algorithm. Inter-click interval was the parameter that ranked second in contribution for both the click and joint analyses. Therefore, ICI should be carefully considered, since it depends on the distance between the target and the individual, which waits for the return of an echo before it emits the next click [36]. With the exception of the 3 dB bandwidth and ICI, the rank contribution of the other parameters changed depending on the type of analysis. Delta frequency had zero splits in whistle analysis, while in the joint step this parameter was third in rank contribution. Therefore, the importance of a variable may pertain only to its performance in the tree in question; it cannot necessarily be generalised to the performance of the same variable in any other tree [14].

Based on the DFA, killer whales, rough-toothed dolphins and Atlantic spotted dolphins had the most distinctive whistles, whereas the tree algorithm model showed this for bottlenose dolphins. For click discrimination, short-beaked common dolphins and Risso’s dolphins had the highest correct classification in the DFA, and the tree model showed that for bottlenose dolphins and Risso’s dolphins. When whistles and clicks were joint classified in the DFA, short-beaked common dolphins, Risso’s dolphins and killer whales had 100% correct classification; while in the tree model, short-beaked common dolphins, long-finned pilot whales and bottlenose dolphins showed the highest classification scores. Therefore, these differences may be because DFA is a parametric method for classification where it uses several assumptions (see “Discriminant function analysis” in the “Statistical analysis” section). By contrast, the use of classification trees is a non-parametric technique that neither assumes any specific distribution of the data nor is influenced by outliers; furthermore, trees are tolerant of observations with missing values [37,38].

Considering the limited number of encounters for each species, the acoustic data presented in this work represent only a portion of the acoustic repertoire and associated behavioural contexts and should not be extrapolated for all population in the South Atlantic or other basins. Small sample sizes may increase the classification accuracy especially when prior probabilities were calculated by from sample size [39]. In order to minimize sample size influence in the DFA, the non–parametric classification tree were applied as complementary method and equal probability was assign in the DFA. Also, since vocalizations were manually selected, the classification performance for automatic detected signals should be investigated. Therefore, the present findings are preliminary, requiring further investigation on a broader and diverse dataset.

## Conclusions

The eight delphinid species, recorded on the outer continental shelf and slope of the western South Atlantic Ocean, showed overall species-specific qualities in their whistles and clicks. When taken individually, echolocation clicks were more efficient in distinguishing species; this may be related to the behavioural context encoded in whistles, the relationship between echolocation signal features, and an animal’s head morphology, particularly the organs associated with the production of sound, which make it feasible to accurately determine a species by its clicks. However, analysing both signals in combination enhanced the correct classification scores. An integrative approach potentially improves the classification process, once it considers the different signals produced by the species as part of a whole bioacoustics system employed in different ecological contexts. However, it is important to highlight that our findings were resulted from a limited database, especially for whistles. Future work should further investigate the effect of behaviour on whistle classification systems by including more data, which would allow accessing the variability of species instead of individual variability. Additionally, future comparisons among acoustic recordings of the same species in different geographic regions may provide information that could be used to elucidate phylogenetic and evolutionary patterns [18], particularly when associated to morphological and genetic aspects, and to investigate inter- and intra-population variations based on differences observed in their acoustic parameters.

## Supporting information

S1 Fig Spectrograms of clicks trains, Y-axis: Frequency (kHz) and X-axis: Time (ms). (a) Shows a train that could be aurally assigned to one vocalizing animal and did not show any other clicks belonging to a different train. (b) Overlapped clicks that were not considered for ICI measurements.
(TIFF)

S1 Table Discrimination of whistles and clicks parameters for all species by Kruskal-Wallis one-way analysis of variance. Values are given as Chi^2^ test results and their p-values. Significant values (p<0.05) in bold. For post-hoc Dunn test only significant differences are shown. MinF: minimum frequency, MaxF: maximum frequency, DeltaF: delta frequency, PeakF: peak frequency, CentreF: centre frequency, BeginF: beginning frequency, EndF: ending frequency, Duration, ICI: inter-click interval, 3 dB BW: 3 dB bandwidth, 10 dB BW: 10 dB bandwidth.
(XLSX)

S2 Table Whistles discriminant scores report providing the predicted classification of each observation. The columns are: species; Distance: Mahalanobis distance from an observation to group for the classification; Prob: estimated probability of the observation’s actual classification; -Log(prob): negative of the log of probability (large values identify observations that are poorly predicted in terms of membership in their actual categories); Predicted: Predicted classification of the observation; Prob (pred): estimated probability of the observation’s predicted classification. *indicates misclassified observations.
(XLS)

S3 Table Clicks discriminant scores report providing the predicted classification of each observation. The columns are: species; Distance: Mahalanobis distance from an observation to group for the classification; Prob: estimated probability of the observation’s actual classification; -Log(prob): negative of the log of probability (large values identify observations that are poorly predicted in terms of membership in their actual categories); Predicted: Predicted classification of the observation; Prob (pred): estimated probability of the observation’s predicted classification. *indicates misclassified observations.
(XLS)

S4 Table Discriminant scores report of joint analysis providing the predicted classification of each observation. The columns are: species; Distance: Mahalanobis distance from an observation to group for the classification; Prob: estimated probability of the observation’s actual classification; -Log(prob): negative of the log of probability (large values identify observations that are poorly predicted in terms of membership in their actual categories); Predicted: Predicted classification of the observation; Prob (pred): estimated probability of the observation’s predicted classification. *indicates misclassified observations.
(XLS)
