# Supplementary figures and images for: Integrative bioacoustics discrimination of eight delphinid species in the western South Atlantic Ocean
Source: PLoS One. 2019 Jun 6;14(6):e0217977. doi: 10.1371/journal.pone.0217977 (PMC6553770; doi:10.1371/journal.pone.0217977)

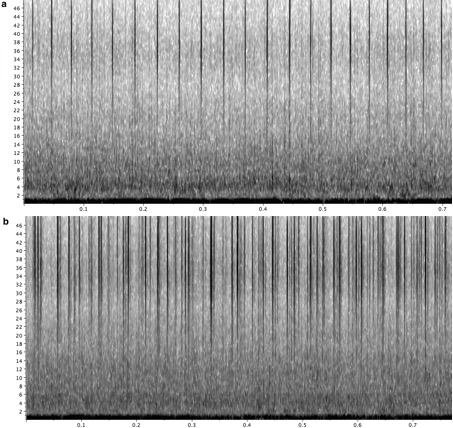

Supplement: S1 Fig — (a) Shows a train that could be aurally assigned to one vocalizing animal and did not show any other clicks belonging to a different train. (b) Overlapped clicks that were not considered for ICI measurements. (TIFF) [file pone.0217977.s001.tiff]
